# Rac inhibits thrombin-induced Rho activation: evidence of a Pak-dependent GTPase crosstalk

**DOI:** 10.1186/1750-2187-1-8

**Published:** 2006-12-06

**Authors:** Hans Rosenfeldt, Maria Domenica Castellone, Paul A Randazzo, J Silvio Gutkind

**Affiliations:** 1 Oral and Pharyngeal Cancer Branch, National Institute of Dental and Craniofacial Research, National Institutes of Health, DHHS, Bethesda, Maryland, 20892-4330, USA; 2 Laboratory of Cellular Oncology, CCR, National Cancer Institute, National Institutes of Health, DHHS, Bethesda, Maryland, 20892-4340, USA

## Abstract

The strict spatio-temporal control of Rho GTPases is critical for many cellular functions, including cell motility, contractility, and growth. In this regard, the prototypical Rho family GTPases, Rho, Rac, and Cdc42 regulate the activity of each other by a still poorly understood mechanism. Indeed, we found that constitutively active forms of Rac inhibit stress fiber formation and Rho stimulation by thrombin. Surprisingly, a mutant of Rac that is unable to activate Pak1 failed to inhibit thrombin signaling to Rho. To explore the underlying mechanism, we investigated whether Pak1 could regulate guanine nucleotide exchange factors (GEFs) for Rho. We found that Pak1 associates with P115-RhoGEF but not with PDZ-RhoGEF or LARG, and knock down experiments revealed that P115-RhoGEF plays a major role in signaling from thrombin receptors to Rho in HEK293T cells. Pak1 binds the DH-PH domain of P115-RhoGEF, thus suggesting a mechanism by which Rac stimulation of Pak1 may disrupt receptor-dependent Rho signaling. In agreement, expression of a dominant-negative Pak-Inhibitory Domain potentiated the activation of Rho by thrombin, and prevented the inhibition of Rho by Rac. These findings indicate that Rac interferes with receptor-dependent Rho stimulation through Pak1, thus providing a mechanism for cross-talk between these two small-GTPases.

## Background

Rho family GTPases control a wide variety of critical cell functions that are important in development, immunity, and disease processes ranging from oncogenesis, hypertension, and asthma. These cell behaviors include migration, proliferation, contact-inhibition, and contraction [1]. Proper execution of these cellular processes depends on the fine spatio-temporal regulation of small GTPases within a cell. This is achieved by the coordinated regulation of guanidine exchange factors (GEFs), which stimulate Rho proteins, and GTPase activating proteins (GAPs) and GDP dissociation inhibitors (GDIs), that terminate or inhibit their function [2]. The vast majority of GEFs contain a Dbl-homology (DH) domain, by which they bind and stabilize small GTPases in a nucleotide-free state, and use the 10-fold molar excess of intracellular GTP over GDP to promote their subsequent binding to GTP [2]. GAP proteins, on the other hand, exert a negative regulation by associating with GTP-bound Rho proteins and enhancing their GTPase activity. GDIs also inhibit Rho GTPase activity, by binding inactive GDP-bound proteins, and stabilizing Rho GTPases in this "off" state [3]. Of these small GTPase regulators, DH-containing GEF proteins have been the best described mediators of signaling from cell surface receptors, including G protein coupled receptors and receptor tyrosine kinases, to the small GTPases [4].

The changes in overall cell behavior resulting from the activation of Rho GTPases by GEFs are reflected in a series of well-described morphological alterations, including the formation of actin-filled protrusions such as filapodia and lammellipodia, and the assembly of parallel actin cables (stress-fibers) [1,5]. Among these cellular structures, the Rho-dependent formation of stress-fibers has been implicated in the contractility of cultured fibroblasts [6]. Motile cells polarize during chemotaxis into leading and trailing edges that are characterized by Cdc42 and Rac activity at the leading edge and Rho activity at the retracting end of the cell [7]. The presence of a retracting subcellular compartment playing counterpoint to the lammellipodia-rich leading edge is a hallmark feature of the polarized and motile cell. Inhibition of Rho-dependent signals with pharmacological inhibitors or dominant negative mutants, destroys this polarization, producing impaired chemotactic responses [7].

Since the initial description of the proto-typical Rho family GTPases, Rho, Rac, and Cdc42, it has been recognized that these molecules regulate each other's activity. In Swiss 3T3 cells, the effects of over-active Rho, Rac, and Cdc42 mutants on actin cytoskeleton morphology suggest a cascade where Cdc42 activates Rac, which in turn activates Rho [5]. More recent studies using other cell types, however, have shown that Rho and Rac can have opposing effects on cell migration and growth cone extension [7-10]. In line with the latter, we found that Rac prevents Rho activation and stress fiber formation in response to thrombin in both endothelial cells and HEK293T cells. While exploring the underlying mechanism, we observed that Rac effector domain mutants that do not to bind Pak1, a Rac effector, fail to inhibit thrombin-induced stress fiber formation and Rho activation. As thrombin can stimulate Rho through the α subunits of heterotrimeric G proteins of the G_12/13 _family, we examined whether Pak1 could interact with members of the RGS-containing family of Rho GEF, PDZ-RhoGEF, LARG and P115RhoGEF, which act downstream from Gα_12/13 _[11,12]. Indeed, we found that Pak1 can bind the DH-PH domain of P115RhoGEF, thereby forming a molecular complex *in vivo*. Furthermore, we provide evidence that Pak1 may represent a point of signal integration by enabling Rac to inhibit receptor-dependent Rho stimulation.

## Results

### Rac inhibits thrombin-induced stress fiber formation and Rho activation

**Figure 1:**
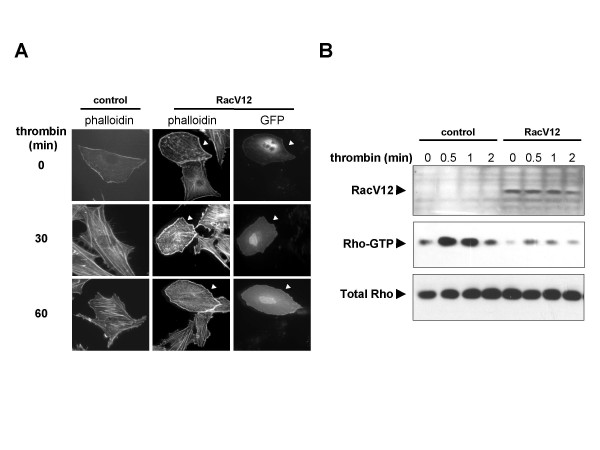
Activated Rac inhibits thrombin signaling to Rho. **(A) **PAE cells transfected with pCEFL-GFP as a control or with the activated RacV12 mutant and pCEFL-GFP together (RacV12) were starved overnight on poly-D-lysine coated glass cover-slips, treated with thrombin 5 U/ml for indicated times, fixed, and stained with TRITC-phalloidin. Arrows point to transfected cells as judged by co-transfection with pCEFL-GFP. **(B) **HEK293T cells were transfected with pCEFL-GFP as a control or with the activated RacV12 mutant and pCEFL-GFP together with RacV12 (RacV12), cultured overnight, starved for 4 h, and treated with thrombin for indicated times. Fresh lysates were used for Rho-pulldown assays with GST-Rhotekin to detect relative amounts of Rho-GTP. Total Rho levels were detected by western blot in total cell lysates as a loading control.

In order to investigate the interplay between Rho and Rac signaling, we tested whether thrombin-dependent stress-fiber formation was sensitive to Rac signaling (Figure 1A). Incubation of endothelial cells with thrombin showed striking increases in stress-fibers. These structures appeared as thick actin cables running along the length of the cell. Instead, Rac-transfected cells took on an invariant rounded shape, exhibited high levels of cortical actin, and did not form stress-fibers upon thrombin stimulation. These results suggested that Rac signaling strongly affected the ability of thrombin to stimulate Rho. To investigate this contention, we asked whether the presence of constitutive Rac signaling modulated the intracellular levels of Rho bound to GTP. As shown in figure 1B, transfection of an active form of Rac in HEK293T cells prevented the accumulation of Rho-GTP in response to thrombin stimulation. Taken together, these data revealed that Rac signaling abrogates thrombin signaling to Rho.

**Figure 2:**
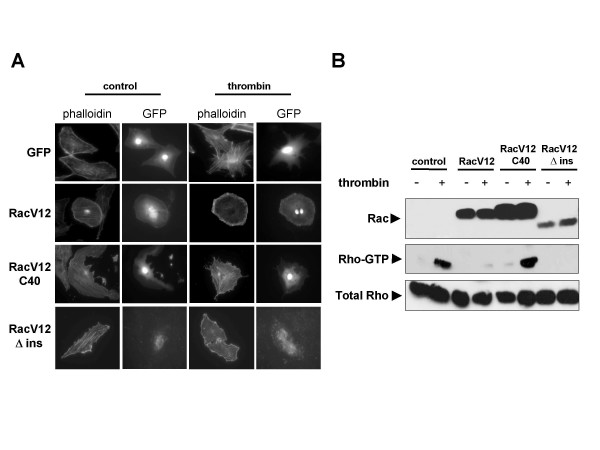
Activated Rac requires Pak1 binding for inhibition of thrombin signaling to Rho. **(A) **PAE cells transfected with pCEFL-GFP as a control or with RacV12, RacV12C40, RacV12Δins mutants together with pCEFL-GFP as indicated were starved overnight on poly-D-lysine coated glass cover-slips, placed in serum-free media (control) or treated with thrombin (5 U/ml) 30 min, fixed and stained with TRITC-labelled phalloidin. Transfected cells were detected by GFP flourescence. **(B) **HEK293T cells were transfected with pCEFL-GFP as a control or with the RacV12 mutants shown in (A) together with pCEFL-GFP, cultured overnight, starved 4 h, treated with thrombin (5 U/ml) for 1 min, and lysed. Fresh lysates were used for Rho-pulldown assays with GST-Rhotekin to detect relative amounts of Rho-GTP. Total Rho levels were detected by western blot in total cell lysates as a loading control.

To further examine which signaling pathways downstream of Rac might be inhibiting thrombin signaling to Rho, we took advantage of the availability of activated Rac mutants harboring additional mutations that restrict their signaling ability. As shown in figure 2, a RacV12 mutant missing the insert region of the Rac1-α domain that is implicated in Rac-dependent activation of reactive oxygen species (ROS) was nevertheless effective in blocking stress-fiber formation stimulated by thrombin [13]. By contrast, RacV12C40, an effector domain mutant of Rac that does not bind nor activate Pak, was unable to prevent stress-fiber formation in response to thrombin (Figure 2A) [14]. We confirmed these results with Rho-pulldown assays to detect relative levels of Rho-GTP after thrombin stimulation. Both control cells and cells transiently over-expressing RacV12C40 accumulated similar levels of Rho-GTP after thrombin stimulation (Figure 2B). However, both the RacV12 and RacV12Δins mutants prevented thrombin treatment from increasing Rho-GTP levels, paralleling the effects seen with stress-fiber formation.

### Pak1 interacts with P115RhoGEF

**Figure 3:**
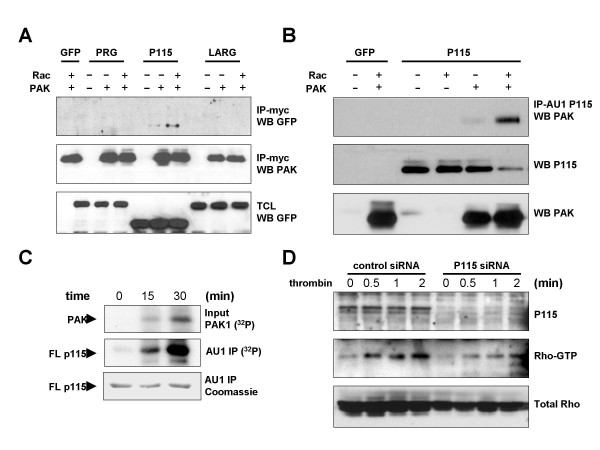
Pak1 binds and phosphorylates P115RhoGEF. **(A) **HEK293T cells were transfected with expression plasmids encoding GFP, GFP-PDZ-RhoGEF (PRG), GFP-P115RhoGEF (P115), or GFP-LARG (LARG) in the presence or absence of wild-type myc tagged Pak1 and/or the activated Rac mutant RacQL (Rac). Lysates were immunoprecipitated with anti-myc antibody, and immunoprecipitates tested for GEF content by western blotting with anti-GFP antibody. **(B) **HEK293T cells were transfected with expression plasmids encoding GFP, GFP-GFP-P115RhoGEF (P115) in the presence or absence of wild-type myc tagged Pak1 and/or the activated Rac mutant RacV12 (Rac). Lysates were incubated with anti-AU1 antibody, and immunoprecipitates tested for Pak1 content by western blotting with anti-Pak1 antibody. (**C) **HEK293T cells were transfected with AU1-tagged full length P115RhoGEF. Two days post-transfection, cells were harvested in lysis buffer. Lysates were incubated with anti-AU1 antibody and immunoprecipitates were placed in kinase buffer containing recombinant Pak1 and γ-^32^P-ATP for times indicated. Kinase reactions were stopped with the addition of sample buffer and boiling. Samples were resolved by SDS-PAGE and acrylamide gels were transferred to PVDF membranes. Resulting blots were exposed to X-ray film 24 h to detect phosphorylated proteins. Total immunoprecipitated P115RhoGEF levels were assessed by coomassie staining. **(D) **HEK293T cells were transfected with control or P115RhoGEF-specific siRNA oligonucleotides, and treated with thrombin for times indicated. Fresh lysates were used for Rho-pulldown assays with GST-Rhotekin to detect relative amounts of Rho-GTP. Total Rho levels were detected by western blot in total cell lysates as a loading control.

As RacV12C40 mutant is unable to bind Pak1, we therefore focused on whether Pak1 might be involved in Rac inhibition of thrombin signaling to Rho. Rho activation stimulated by G_12/13 _coupled receptors such as the thrombin receptors has been shown to occur through the RGS-containing Rho GTP exchange factors, a protein family that includes PDZ-RhoGEF, LARG, and P115RhoGEF [12,14,15]. Interestingly, in a recent study we have observed that Pak4, but not Pak1 and Pak2, inhibits the functional activity of PDZ-RhoGEF upon binding to its C-terminal regulatory region [16]. Based on this observation, we investigated whether there was a physical interaction between RGS-containing RhoGEFs and Pak1. As shown in figure 3A, P115RhoGEF, but not PDZ-RhoGEF or LARG, co-immunoprecipitated with Pak1, and this interaction was enhanced by the presence of activated Rac. Molecular association between Pak1 and P115RhoGEF could also be detected when P115RhoGEF was immunoprecipitated (Figure 3B). Moreover, immunoprecipitated full length P115RhoGEF was efficiently phosphorylated by Pak1 (Figure 3C).

**Figure 4:**
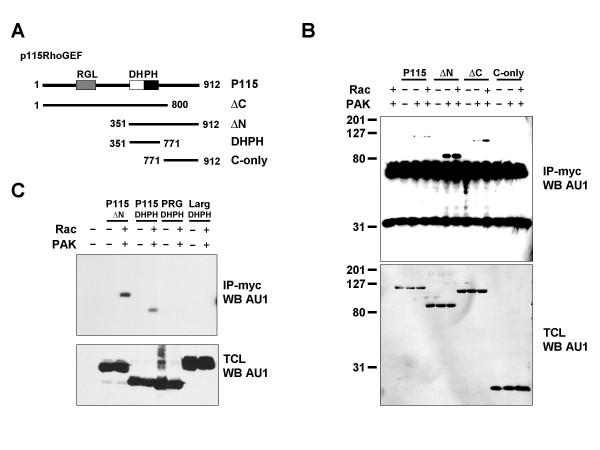
Pak1 binds the DH-PH domain of P115RhoGEF. **(A) **Constructs used in these experiments. **(B) **HEK293T cells were transfected with expression plasmids encoding the AU1-tagged constructs shown in (A) in the presence or absence of wild-type myc-tagged Pak1 and/or the activated Rac mutant RacQL (Rac). Lysates were immunoprecipitated with anti-myc antibody, and immunoprecipitates tested for GEF content by western blotting with anti-AU1 antibody. **(C) **HEK293T cells were transfected with expression plasmids encoding AU1-tagged DH-PH domains of P115RhoGEF (P115), PDZ-RhoGEF (PRG), and LARG (LARG) in the presence or absence of wild-type myc tagged Pak1 and/or the activated Rac mutant RacQL (Rac). The P115RhoGEF ΔN construct is included as a control. Lysates were immunoprecipitated with anti-myc antibody, and immunoprecipitates tested for GEF content by western blotting with anti-AU1 antibody.

To assess the contribution of P115RhoGEF in thrombin signaling to Rho, we used siRNA oligonucleotides to knockdown P115RhoGEF in HEK293T cells (Figure 3D). Depletion of P115RhoGEF in these cells greatly impaired Rho activation in the presence of thrombin. Although P115RhoGEF knockdown decreased total Rho levels slightly, quantitation of ECL exposures from these experiments using NIH Image™ showed that P115RhoGEF knockdown inhibited >60% of Rho-GTP accumulation in response to thrombin treatment. To further define the interaction between P115RhoGEF, we used a set of P115RhoGEF deletion constructs to determine where Pak1 binds (Figure 4A). Co-immunoprecipitation experiments with these mutants revealed that deletion of the P115RhoGEF N-terminus greatly facilitated binding to Pak1 (Figure 4B). By contrast, deletion of the C-terminus produced a protein that was still regulated by Rac. The C-terminal region, which has previously been described as a site of protein-protein interaction in RGS GEFs, was by itself unable to bind Pak1. These results narrowed down the region of interaction between Pak1 and P115RhoGEF to a region between amino acids 351 and 771. Interestingly, these residues form the DH and PH domains of P115RhoGEF. To directly test whether Pak1 could bind this motif, we asked whether Pak1 could bind the DH-PH domain of P115RhoGEF, as well as the isolated DH-PH domains of PDZ-RhoGEF and LARG. Of these three proteins, only the DH-PH domain of P115RhoGEF was able to bind Pak1 (Figure 4C)

### Rac requires Pak1 for Rho Inhibition *in vivo*

**Figure 5:**
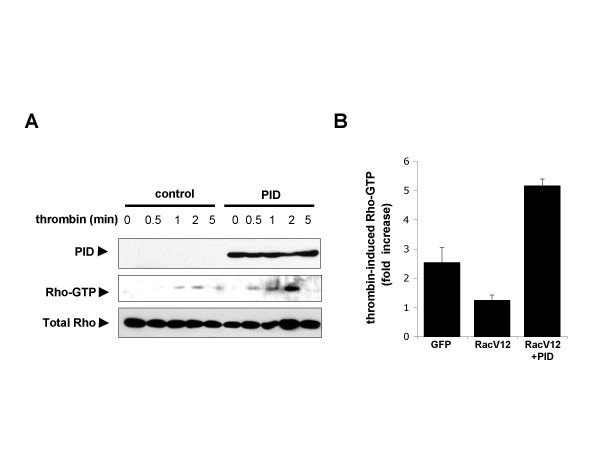
Pak1 inhibitory domain (PID) reverts Rac inhibition of Rho by Rac. (A) HEK293T cells were transfected with plasmids expressing a GFP or GFP-PID fusion protein (PID) (1 μg/plate) and incubated for two days, serum-starved 4 h, and treated with thrombin (5 U/ml) for times indicated. Fresh lysates were used for Rho-pulldown assays with GST-Rhotekin to detect relative amounts of Rho-GTP. Total Rho levels were detected by western blot in total cell lysates as a loading control. GFP western blot from total cell lysate was used to detect the GFP-PID fusion protein. (A) Cells were co-transfected with plasmids encoding the active RacV12 (Rac) mutant in the presence of control pCEFL-GFP or pCEFL-GFP-PID (2 μg/plate). After 2 days incubation cells were serum-starved 4 h, stimulated with thrombin (5 U/ml, 2 min), and assayed for Rho-GTP levels with pulldown assays. Data are expressed as fold increase ± SEM in Rho-GTP with respect to unstimulated control cells.

We next challenged the notion that Pak1 function is necessary for Rac-mediated inhibition of Rho. In resting, unstimulated, cells Pak1 exists as a homodimer in which each binding partner inhibits the kinase domain of the other [17]. The region of Pak1 that binds and inhibits the kinase domain has been isolated. This Pak1 inhibitory domain (PID) can be used to block Pak1 activity and the biological effects of Pak1 signaling, such as the dissociation of Rac from RhoGDI [18,19], GPCR-dependent cell transformation and estrogen-dependent transcription in breast cancer cells [20,21]. Indeed, transfection with this PID domain dramatically increased the Rho response to thrombin (Figure 5A). Importantly, expression of the Pak1 PID domain reversed the ability of Rac signaling to inhibit thrombin activation of Rho (Figure 5B). These data strongly implicated Pak1 as a mediator of Rac inhibition of thrombin signaling to Rho.

## Discussion

The Rac and Rho GTPases have opposing effects in many cell types, including endothelial, smooth muscle, and neuronal cells, and can override signals from each other depending on cell type [8-10,22]. In this study, we show that the ability of thrombin to promote stress fiber formation and stimulate Rho can be inhibited by activated forms of Rac. In search for the underlying mechanism, we found that Rac effector domain mutants that cannot interact with Pak1 fail to block thrombin signaling to Rho. Indeed, inhibition of Pak by a Pak-inhibitory construct enhanced the extent of Rho activation after thrombin treatment, and prevented co-transfected Rac from blocking the increase in Rho-GTP levels. Furthermore, we provide evidence that Rac promotes the formation of a molecular complex between Pak1 and P115RhoGEF *in vivo*, a signaling molecule that is required for the bulk of thrombin signaling to Rho, thus providing a likely mechanism by which Rac can restrict Rho activation.

Recent reports have implicated Pak family kinases in the regulation of Rho GEFs. In particular, a two hybrid screen approach for molecules binding the C-terminal regulatory domain of PDZ-RhoGEF identified Pak4, a Cdc42 effector that is member of the class II Pak family as a binding partner [16,23]. Similarly, GEF-H1, a microtubule-localized Rho Exchange Factor, binds Pak1 at its C-terminus, and GEF-H1 a substrate for this kinase [24]. Moreover, activated Cdc42 can stimulate Pak1 phosphorylation of GEF-H1 *in vitro*, suggesting a possible role for Pak1 in the regulation of GEF-H1 downstream of cellular receptors. Recently, Alberts and colleagues reported the inhibitory phosphorylation of NET1 just outside its DH-PH domain, by Pak1 [25]. Thus, although we cannot rule out that Rac may regulate RhoGDIs or RhoGAPs through Pak1 thereby inhibiting Rho activation by thrombin, our findings suggest that this inhibitory effect of Rac can be exerted through the binding of Pak1 to P115RhoGEF.

The precise mechanism by which Pak1 regulates P115RhoGEF function is at the present unknown. Although Pak1 can bind the isolated DH-PH domain of P115RhoGEF, Pak1 does not phosphorylate this domain when expressed and purified from *E. coli *in *in vitro *kinase assays, nor does the binding of Pak1 affect the *in vitro *GTP-exchange activity of p115RhoGEF on RhoA (not shown). Similarly, we did not observe changes in the *in vitro *Rho GEF activity of P115RhoGEF when its different deletion constructs were expressed together with Pak1 in 293T cells (not shown), which is aligned with the accumulating evidence that suggests that the *in vitro *activity of RGS-containing Rho GEFs does not reflect fully their likely complex regulation *in vivo*. For example, these Rho GEFs are poorly activated *in vitro *by Gα_12_, in spite of the strong evidence that Gα_12 _interacts and activates the GEF activity of this class of Rho GEFs *in vivo *[26,27]. Similarly, the removal of the potent inhibitory activity exerted by the C-terminal region of p115RhoGEF, PDZ-RhoGEF, and LARG *in vivo *does not result in the enhanced ability of these GEFs to stimulate nucleotide exchange on Rho *in vitro *[28]. Thus, it is possible that additional molecules may participate in the regulation of P115RhoGEF by Pak1 *in vivo*, or that Pak1 may interfere with the targeting of p115RhoGEF by activated G_12/13 _to a particular subcellular fraction or membrane microdomain, whose functional consequences would not be reflected by the GEF activity of p115RhoGEF *in vitro*.

On the other hand, the finding that Pak1 binds the DH-PH domain of P115RhoGEF may have important regulatory consequences upon G protein-link receptor activation. Previous studies have shown that the RGS domain of P115RhoGEF regulates the DH-PH catalytic activity [26]. In this model, the RGS domain of P115RhoGEF binds the DH-PH domain intramolecularly, thus hindering sterically its ability to interact with Rho proteins. Pak1 binding to full-length P115RhoGEF is stimulated by the presence of active Rac1, suggesting that activation of Pak1 by Rac enhances the interaction between Pak1 and P115RhoGEF. However, this regulation is abrogated when the N-terminal 351 amino acids of P115RhoGEF, that include the RGS domain, are deleted. Furthermore, the isolated DH-PH domain of P115RhoGEF binds Pak1 constitutively *in vivo*. This may reflect what is likely to occur upon ligand-induced activation of P115RhoGEF. In this scenario, stimulation of GPCRs, such as thrombin receptors, activates Gα_12/13 _subunits which will then bind the RGS domain of P115RhoGEF de-repressing and exposing its DH-PH domain, thereby stimulating Rho proteins [27]. This exposed DH-PH domain can then be more accessible for its interaction and inactivation by Pak1, if Rac is concomitantly activated. Indeed, many receptors are coupled to both the G_12/13 _and G_i _pathways; therefore it is possible that the same receptor may activate Rho through G_12/13 _and Rac through Gi, the latter regulating the temporal and spatial activation of Rho.

In this regard, although the underlying mechanisms of crosstalk between signaling from cellular receptors remain elusive, there is a growing literature describing the interactions between G_12/13 _and G_i _signaling systems and the necessity for this co-regulation in the proper execution of cell behaviors such as cell migration and neurite extension [7]. For example, the bacterial peptide formyl-methionyl-leucine-phenylalanine (fMLP) is a strong chemoattractant for neutrophils [29]. This molecule binds a G_i _coupled receptor, and activates Gi-dependent pathways such as Rac, PI-3-Kinase, and AKT in neutrophils and cells that resemble this cell-type such as differentiated HL-60 cells [30,31]. However, Xu et al. have recently shown that fMLP also stimulates Rho in these cells, and that pertussis toxin treatment fails to inhibit this Rho activation, while totally shutting down signaling to Rac [7]. Thus, the fMLP receptor is coupled to both G_12/13 _and G_i _in HL60 cells. These findings lead to a model in which a single receptor uses dual G_i _and G_12/13 _coupling to create a polarized migrating cell. Crosstalk between G_12/13 _and G_i _can also occur when multiple receptor subtypes exhibiting distinct coupling ability are activated by their ligand, such as those for lysophosphatidic acid (LPA) and sphingosine-1-phosphate (S1P) that are preferentially coupled to G_i_, G_12_/G_13 _and G_i_, or G_q _[32], and can be also activated indirectly by other cell surface receptors, such as receptor tyrosine kinases [33,34]. These examples of G_12/13 _and G_i _co-regulation of the same cell behavior highlight the necessity of these two pathways to modulate each other, and Pak1/P115RhoGEF complex formation may provide such a link.

## Conclusion

The finding that Rac regulates the association of Pak1 with P115-RhoGEF may represent a potential mechanism of cross-talk among Rho GTPases, in which the effector of one Rho GTPase regulates the function of a GEF for another small GTP-binding protein. Indeed, this appears to be an emerging theme in cell biology, as documented by recent studies demonstrating the de-repression of p190RhoGAP by a ROS-mediated mechanisms stimulated by the persistent and prolonged expression of active Rac, the negative regulation of Rac-mediated ROS formation by Cdc42, the interaction between Pak4 and PDZ-RhoGEF, and the interaction between Pak1 and GEF-H1 and NET1 [16,22,24,25,35]. Considering the importance of the proper coordination of Rho GTPase function for many key developmental and physiological processes [4], we can expect that the full elucidation of the mechanism underpinning the coordinated activation/inactivation of Rho GTPases may ultimately help explain how the dysregulated activity of Rho proteins can contribute to human diseases.

## Methods

### Materials

Bovine serum albumin (fatty acid-free) Dulbecco's modified Eagle's medium (DMEM), trypsin/EDTA solution, and Fetal bovine serum (FBS) were purchased from Sigma (St. Louis, Mo); anti-mouse secondary HRP-linked antibody was from ICN pharmaceuticals (Aurora, Ohio); and Glutathione-sepharose beads were purchased from Amersham Biosciences (Piscataway, NJ). Anti-GFP, AU5, and AU1 antibodies were purchased from Covance (Princeton, NJ). The Anti-Rac1 antibody was purchased from BD Transduction Laboratories (Franklin Lakes, NJ).

### DNA constructs

pCMV6M-Pak1 was kindly provided by J. Field [36]. pCEFL-AU1-P115RhoGEF and deletion constructs were subcloned as previously described [28]. The Rac^Q61L^, Rac^G12V^, Rac^G12V, Y40C ^mutants were generated using PCR-based mutagenesis (Stratagene, La Jolla, CA) and subcloned into pCEFL-AU5. The Rac^V12Δ ^ins mutant was constructed by deleting amino acids 124–135 which comprise the insert region of Rac by using an overlapping primer extension approach [37]. The Pak1 inhibitory domain encoding amino acids 67–147 of Pak1 was amplified by PCR and subcloned into pCEFL-GFP. The P115RhoGEF DH-PH sequence encoding amino acids 351–771 was transferred as BglII/NotI fragments into pGEX-4T3 generating the pGEX-P115RhoGEF DH-PH clone that was expressed in *E. coli*.

### Immunohistochemistry

Porcine airway endothelial (PAE) cells were grown in 6-well plates on coverslips and co-transfected with pCEFL-EGFP and pCEFL-AU5-Rac constructs. Cells were serum starved for 16 hours and then stimulated with thrombin (5 U/ml) (Sigma-Aldrich, Inc; St. Louis, MO), washed twice with phosphate buffered saline (PBS), fixed with 3.7% paraformaldehyde in PBS and then permeabilized with PBS containing 0.5% Triton X-100. Following 30 minutes incubation in PBS containing 1% BSA, cells were stained with TRITC-phalloidin (Invitrogen Molecular Probes, Carlsbad, CA) following the manufacturer's instructions. Coverslips were mounted using Vectashield mounting medium for fluorescence (Vector Laboratories, Burlingame, CA) and visualized using Axioplan2 microscope (Carl Zeiss MicroImaging, Inc, Thornwood, NY). GFP and TRITC fluorescence was detected using Chroma Technology (Rockingham, VT) filter sets and 41001 and 41002B, respectively.

### Transfection, Immunoprecipitations and Western blotting

Human embryonic kidney 293T (HEK293T) cells were maintained in Dulbecco's modified Eagle's medium supplemented with 10% fetal bovine serum. Porcine airway endothelial (PAE) cells were cultured in F12 media containing 10% fetal bovine serum. Transfections were performed in 60 mm or 100-mm cell culture dishes using LipofectAMINE Plus™ reagent (Invitrogen, Carlsbad, CA) according to the manufacturer's protocol. The total amount of DNA was adjusted to 3 μg/plate with pCEFL-EGFP when necessary. Transfection of siRNA oligonucleotides was achieved using the Hyperfect™ reagent (Qiagen, Valencia, CA) according to the manufacturer's instructions. The P115RhoGEF sense and antisense siRNA oligonucleotides were r(GCA GCU CUG AGA ACG GCA A)dTdT and r(UUG CCG UUC UCA GAG CUG C)dTdG, respectively. Sense and antisense negative control siRNA oligonucleotides were r(UUC UCC GAA CGU GUC ACG U)dT dT and r(ACG UGA CAC GUU CGG AGA A)dT dT, respectively. Immunoprecipitations and Western blotting were performed as previously described [38].

### Rho-pulldown Assays

Cytosolic extracts were prepared from HEK293T cells transfected with vector or expression plasmids. Rho-pulldown assays using a GST-Rhotekin fusion protein were performed essentially as previously reported [39].

### Kinase Assays

Pak1 autophosphorylating, MBP phosphorylating and P115RhoGEF DH-PH phosphorylating kinase activity was assessed in vitro, essentially as described previously for extracellular signal-regulated kinase assays with a different kinase buffer (12.5 mM MOPS, pH 7.5, 1 mM beta-glycerophosphate, 10 mM MgCl_2_, 3 mM MnCl_2_, 0.5 mM NaF, 0.5 mM ortho-vanadate, 0.5 mM EGTA, 3 mM DTT, 20 μM ATP, 250 μCi/ml ATP γ^32^P) [40].

### Nucleotide Exchange Assays

Rho GTP exchange reactions to measure the catalytic activity of the P115RhoGEF DH-PH were performed in 1× Kinase buffer supplemented with GTPγS and recombinant His-Rho (12.5 mM MOPS, pH 7.5, 1 mM beta-glycerophosphate, 10 mM MgCl_2_, 3 mM MnCl_2_, 0.5 mM NaF, 0.5 mM ortho-vanadate, 0.5 mM EGTA, 3 mM DTT, 20 μM ATP, 4 μM GTPγS, 2 μCi/ml GTPγ^35^S 1 μM His-Rho) in a final volume of 70 μl. At t = 0 1 1 μM of GST-P115RhoGEF DH-PH +/- 0.5–3 μM GST-Pak1 was added to experimental samples and tubes were moved to 30°C water bath. After each time point, 10 μl from each reaction was added to 2 ml cold wash solution (100 mM Tris, pH 7.5, 20 mM NaCl, 1 mM MgCl_2_, 0.5 mM DTT, 1 mg/ml BSA). Each sample was vacuum filtered over nitrocellulose and rinsed 4× with 2 ml wash solution. Filters were placed in 20 ml scintillation fluid (Cytoscint, ICN) and ^35^S activity was determined in a Beckman LSC 6000C Scintillation counter.
